# Symptom management care pathway adaptation process and specific adaptation decisions

**DOI:** 10.1186/s12885-023-10835-0

**Published:** 2023-04-17

**Authors:** Emily Vettese, Farha Sherani, Allison A. King, Lolie Yu, Catherine Aftandilian, Christina Baggott, Vibhuti Agarwal, Ramamoorthy Nagasubramanian, Kara M. Kelly, David R. Freyer, Etan Orgel, Scott M. Bradfield, Wade Kyono, Michael Roth, Lisa M. Klesges, Melissa Beauchemin, Allison Grimes, George Tomlinson, L. Lee Dupuis, Lillian Sung

**Affiliations:** 1 Program in Child Health Evaluative Sciences, Peter Gilgan Centre for Research and Learning, The Hospital for Sick Children, 686 Bay Street, Toronto, ON M5G 0A4 Canada; 2 Driscoll Children’s Hospital, Cancer and Blood Disorders Center, 3533 S. Alameda Street, Corpus Christi, TX 78411 US; 3 Washington University School of Medicine, 660 S Euclid Ave, St Louis, MO 63110 US; 4 Louisiana State University Health Sciences Center/Children’s Hospital, 200 Henry Clay Avenue, New Orleans, LA 70118 USA; 5 Stanford University, 800 Welch Road, Palo Alto, CA 94304 US; 6 Nemours Children’s Hospital of The Nemours Foundation, 6535 Nemours Parkway, Orlando, FL 32827 US; 7 Roswell Park Comprehensive Cancer Center, University at Buffalo Jacobs School of Medicine and Biomedical Sciences, 665 Elm St., Buffalo, NY 14203 US; 8 Cancer and Blood Disease Institute, Children’s Hospital Los Angeles, 4650 Sunset Blvd, Los Angeles, CA 90027 US; 9 Nemours Children’s Health, 807 Children’s Way, Jacksonville, FL 32207 US; 10 Kapi’olani Medical Center for Women & Children, 1319 Punahou Street, Honolulu, Hawai’i 96826 US; 11 Division of Pediatrics, University of Texas MD Anderson Cancer Center, 1515 Holcombe Blvd, Houston, TX 77030 US; 12 Division of Public Health Sciences, Washington University School of Medicine, 600 So Taylor Ave, St. Louis, MO 63110 US; 13 Columbia University School of Nursing/Herbert Irving Cancer Center, 560 West 168th Street, New York, NY 10032 USA; 14 Pediatric Hematology Oncology, University of Texas Health, The Science Center at San Antonio, 7703 Floyd Curl Drive, San Antonio, TX 78229 US; 15 Department of Medicine, Toronto General Hospital, 200 Elizabeth Street, Toronto, ON M5G 2C4 Canada; 16 Department of Pharmacy, The Hospital for Sick Children, 555 University Avenue, Toronto, ON M5G 1X8 Canada; 17 Division of Haematology/Oncology, The Hospital for Sick Children, 555 University Avenue, Toronto, ON M5G 1X8 Canada; 18 Texas A&M University, College Station, TX US; 19 Leslie Dan Faculty of Pharmacy, University of Toronto, Toronto, ON M5S 3M2 Canada

**Keywords:** Symptom management, Care pathway, Supportive care, Pediatric, Oncology, Clinical practice guidelines, Implementation

## Abstract

**Background:**

There is substantial heterogeneity in symptom management provided to pediatric patients with cancer. The primary objective was to describe the adaptation process and specific adaptation decisions related to symptom management care pathways based on clinical practice guidelines. The secondary objective evaluated if institutional factors were associated with adaptation decisions.

**Methods:**

Fourteen previously developed symptom management care pathway templates were reviewed by an institutional adaptation team composed of two clinicians at each of 10 institutions. They worked through each statement for all care pathway templates sequentially. The institutional adaptation team made the decision to adopt, adapt or reject each statement, resulting in institution-specific symptom management care pathway drafts. Institutional adaption teams distributed the 14 care pathway drafts to their respective teams; their feedback led to care pathway modifications.

**Results:**

Initial care pathway adaptation decision making was completed over a median of 4.2 (interquartile range 2.0-5.3) weeks per institution. Across all institutions and among 1350 statements, 551 (40.8%) were adopted, 657 (48.7%) were adapted, 86 (6.4%) were rejected and 56 (4.1%) were no longer applicable because of a previous decision. Most commonly, the reason for rejection was not agreeing with the statement (70/86, 81.4%). Institutional-level factors were not significantly associated with statement rejection.

**Conclusions:**

Acceptability of the 14 care pathways was evident by most statements being adopted or adapted. The adaptation process was accomplished over a relatively short timeframe. Future work should focus on evaluation of care pathway compliance and determination of the impact of care pathway-consistent care on patient outcomes.

**Trial registration:**

clinicaltrials.gov, NCT04614662. Registered 04/11/2020, https://clinicaltrials.gov/ct2/show/NCT04614662?term=NCT04614662&draw=2&rank=1.

**Supplementary Information:**

The online version contains supplementary material available at 10.1186/s12885-023-10835-0.

## Background

Supportive care for pediatric oncology patients focuses on preventing and managing cancer- and treatment-related toxicities. Early assessment and intervention for these toxicities are important as they are associated with morbidity, reduced quality of life, increased healthcare utilization and treatment-related mortality [1]. In general, there is substantial heterogeneity in the supportive care delivered between and within institutions [2, 3]. To increase the consistency of high-quality evidence-based supportive care delivery, it is important to determine optimal treatment approaches for each condition.

Clinical practice guidelines (CPGs) include statements intended to optimize patient care,[4] and are the basis for translating evidence to clinical practice [5]. CPGs are developed by conducting a systematic review of the literature and by convening an expert guideline panel that weighs the benefits and downsides of different treatment options [6–8]. However, CPGs may be difficult to apply directly to individual patients because of their format and lack of consideration of institution-specific factors. One strategy to bridge the gap between care patients should receive (CPG-consistent care) and care they actually receive is care pathway implementation [9]. Care pathways are documents that explicitly describe best practice with respect to the management of a group of patients and consequently, should be based on CPGs [10]. However, the process to rigorously create CPG-based care pathways and to adapt them for use by individual institutions is not well understood. Typically, institution-specific care pathways are developed using unclear procedures without a formal process to create or evaluate them.

There are several potential advantages of establishing institution-specific, evidence-based care pathways for supportive care. They may improve the consistency of supportive care and increase the likelihood that the supportive care delivered is CPG-consistent. In addition, they may reduce cognitive decision-making burden for individual clinicians by explicitly outlining the available care options. Finally, these documents may have positive impacts on patient outcomes including reducing toxicities and improving quality of life. If clear and achievable processes to create and adapt institution-specific care pathways were developed, it might provide a mechanism to improve evidence-based symptom management across institutions.

We describe procedures performed as a component of a cluster randomized trial of 20 institutions in the United States testing whether a web-based application named Supportive care Prioritization, Assessment and Recommendations for Kids (SPARK) can improve symptom control in newly diagnosed pediatric patients with cancer [11, 12]. The intervention under evaluation included adaptation of symptom management care pathways for institutions randomized to the intervention group. The primary objective was to describe the adaptation process and specific adaptation decisions related to CPG-based symptom management care pathways. The secondary objective was to determine if institutional factors were associated with adaptation decisions.

## Methods

The study was approved by the Research Ethics Board of The Hospital for Sick Children (SickKids), the Western Institutional Review Board and the Institutional Review Boards of each participating institution. For patient participation in the trial, informed written consent was obtained from participants and from the parents/legal guardians of minors (age specified by each institution). Patient outcomes will not be presented in this report. The trial was registered at clinicaltrials.gov (NCT04614662) on 04/11/2020. The methods relevant to this analysis consisted of the development of symptom management care pathway templates for 14 symptoms, adaptation of the care pathway templates at 10 institutions and a survey of institutional characteristics.

### Development of symptom management care pathway templates

This study was focused on the management of the 15 symptoms captured by the Symptom Screening in Pediatrics Tool (SSPedi). SSPedi is a reliable, valid and responsive self-report symptom assessment tool for English, French and Spanish-speaking children and adolescents 8–18 years of age receiving cancer treatments [13, 14]. SSPedi includes the following 15 symptoms that were considered most important to patients and their families: disappointed or sad, scared or worried, cranky or angry, problems thinking, body or face changes, tiredness, mouth sores, headache, other pain, tingling or numbness, throwing up, hunger changes, taste changes, constipation and diarrhea. As headache and other pain both reflect pain, 14 symptom management care pathways were required.

The approach to the systematic identification of applicable CPGs relevant to pediatric cancer supportive care has previously been described in detail [15]. In short, the search for CPGs identified those developed or endorsed by key organizations who focus on methodologically robust (trustworthy) CPGs [16]. If CPGs were not identified using this approach, a systematic review of CPGs was conducted. From the identified (source) CPGs, recommendations appropriate for populating a care pathway were identified.

The care pathway templates for all 14 symptoms were similarly structured and included three sections: prevention, assessment and treatment (see Additional file 1 for an example). The template formatting provided visual cues for statements stemming from conditional recommendations from the source CPGs. These recommendations reflected situations where intervention benefits were closely matched with their downsides or where there was considerable imprecision in the estimates. In these cases, institutions could choose to adopt the statement without change, reject the statement entirely or leave decision making to individual clinicians. The template formatting also provided visual cues for statements stemming from strong recommendations from the source CPGs. These recommendations reflected situations in which the intervention benefits clearly outweighed its downsides or vice versa. Consequently, it was anticipated most institutions would choose to adopt the statement with or without center-specific modifications. Finally, potential resources to provide the care outlined in the statements such as consulting services were listed.

### Institutional adaptation of symptom management care pathway templates

**Fig. 1:**
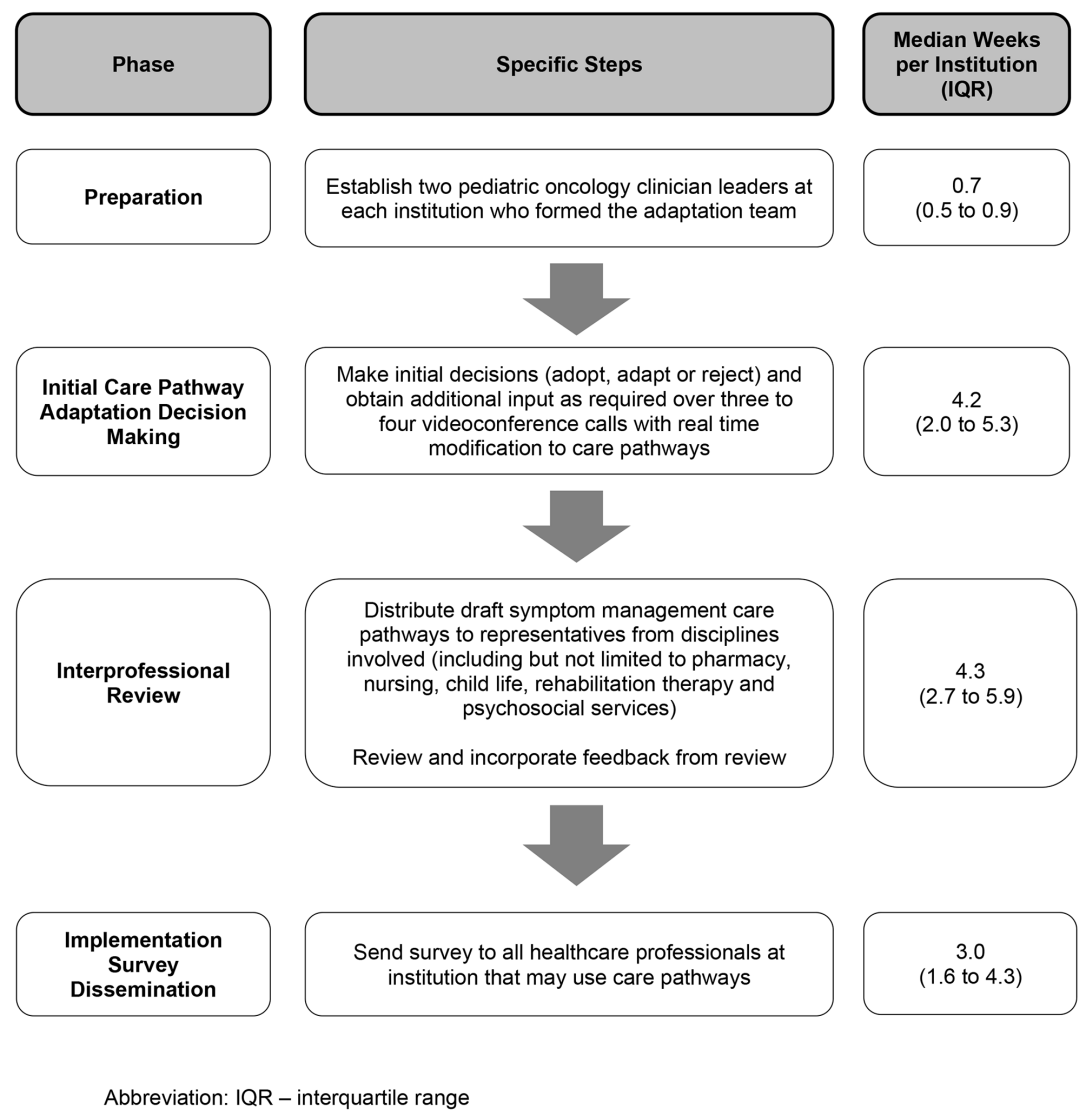
Phases Required to Adapt the 14 Care Pathway Templates at Each of the 10 Institutions

Fig. 1 provides an overview of the adaptation procedures that were executed at each of the 10 institutions randomized to the intervention group. The phases involved were: (1) preparation; (2) initial care pathway adaptation decision making; (3) interprofessional review; and (4) implementation survey dissemination.

(1) Preparation Phase: The preparation phase established two pediatric oncology clinician leaders per institution to undertake the adaptation process, forming the institutional adaptation team. One member was the institutional principal investigator for the cluster randomized trial. The second member was selected by the institutional principal investigator and was a clinician with a particular interest in supportive care and a track record for practice change implementation, where possible.

(2) Initial Care Pathway Adaptation Decision-Making Phase: The second phase comprised the fundamental work of the adaptation process. This phase began with an introductory session describing the process overall and outlining the decision making required by the institutional adaptation team. The structure of the care pathway template was shown including the delineation of formatting reflecting strong vs. conditional recommendations from the source CPGs and formatting signaling that an institutional choice was required. Working through each symptom management care pathway template, we focused on each statement sequentially.

For each statement, the institutional adaptation team made the decision to adopt, adapt or reject the statement regardless of whether the source CPG recommendation was strong or conditional. For statements stemming from conditional recommendations, the institutional adaptation team was encouraged to adapt it to a declarative statement or reject it rather than leave decision making to individual clinicians. Decision making of the institutional adaptation team was not constrained by this encouragement. For statements that were adopted or adapted, the institutional adaptation team framed the statements using language that would be understood by clinicians at that institution including choice of generic or brand name medications. If resources were required such as consulting services, investigations or medications, they described services, investigations or medications in language relevant to their institution. This process included choosing specific medications, in part, based upon those available according to their hospital formulary. The institutional adaptation team could also add statements not included in the care pathway template.

SSPedi measures the degree of bother as reported by the pediatric patient for each symptom. For each treatment section, the institutional adaptation team could more prescriptively direct clinicians to a specific action based upon severity of bother (for example, different actions for severely bothersome symptom compared to mild or moderately bothersome symptom). Conversely, the institutional adaptation team could allow individual clinicians to choose among a list of actions based on severity (for example, listing several actions and directing the user to make their own decision about which actions to undertake based on the degree of bothersome symptoms). Additional file 2 illustrates an example of this choice.

The initial care pathway adaptation decision-making process was accomplished over three to five videoconference calls per institution where the care pathways were revised in real time as the institutional adaptation team made decisions. The calls were recorded. On these calls, the institutional adaptation team could defer decisions using two mechanisms. For decisions that could be addressed rapidly through consulting with local personnel, decisions were made within one week of the call and the resulting statement was drafted or edited and reviewed by email. For decisions that were expected to be more contentious and at the request of the institutional adaptation team, an adaptation panel of key stakeholders could be assembled to debate and achieve consensus for any decision.

Once all 14 symptom management care pathways had been adapted, the lead institution (SickKids) evaluated each of them to ensure consistency in style and language within and across all institutions. Next, we asked the institutional adaptation team to review the draft institutional care pathways as a whole and to make changes if required.

(3) Interprofessional Review Phase: Once each institutional adaptation team approved their 14 draft symptom management care pathways, we asked them to distribute the drafts to key representatives from professions involved in the care pathway actions. These professions included, but were not limited to pharmacy, nursing, child life, rehabilitation services and psychosocial services. Feedback was received and incorporated into the care pathways as determined by the institutional adaptation team. At this point, the care pathways were finalized and up-loaded into SPARK for institution-specific use. Once the care pathways had been finalized, reasons for adaptation and reasons for rejection were categorized by two clinical research associates listening to the audio-recordings. If they disagreed, they discussed until they came to consensus.

(4) Implementation Survey Dissemination Phase: The final phase was an implementation survey sent to all health care professionals at the institution. Institutional principal investigators were given the option to administer it themselves or to provide the names and email addresses to the lead site for distribution. The survey presented the finalized care pathways relevant to the respondent type. For example, physical therapists were shown the constipation, depression, fatigue, pain and peripheral neuropathy care pathways while pharmacists were shown all 14 care pathways. Respondents were asked which care pathways they were most likely to use, which care pathways they were least likely to use, and whether there were any resources that would facilitate implementation of care pathway-consistent care. The purpose of the survey was to raise awareness of the care pathways and to help individualize implementation materials to be created for each institution.

### Survey of institutional characteristics

The creation, distribution and results of the baseline survey have previously been described [17]. In brief, one survey was distributed to all institutions participating in the cluster randomized trial (both intervention and control) and was completed by the institutional principal investigator. Institutional characteristics collected included whether the institution cared for pediatric patients only or both adult and pediatric patients, and patient characteristics such as the number of pediatric patients with cancer diagnosed annually, the percentage of pediatric patients with cancer who were covered by private insurance, public insurance or no insurance, and percentage of patients by sex, race, ethnicity and spoken language. We also asked about the attributes of institutional physicians (medical doctor or doctor of osteopathic medicine), nurse practitioners and physician assistants in terms of number of full-time equivalents on staff and the median number of years in practice working with pediatric oncology patients. Also, the respondent completed a survey regarding baseline (pre-intervention) symptom management CPG or care pathway utilization within their institution.

In addition to these characteristics, we administered the Consolidated Framework for Implementation Research (CFIR) to help understand factors associated with care pathway adaptation decisions [18, 19]. This conceptual framework includes factors that may impact on intervention implementation. As previously described,[17] we only examined inner setting measures, which include culture, culture stress, culture effort, implementation climate, learning climate, leadership engagement and available resources. For analysis, we were most interested in implementation climate as it is more specific to care pathway implementation and it has been identified as being important to influencing implementation in practice [18, 20, 21]. The questions are rated on a 5-point Likert scale consisting of 1=“strongly disagree”; 2=“disagree”; 3=“neutral”; 4=“agree”; and 5=“strongly agree”. Consistent with our previous work, we dichotomized between respondents who agreed (score of 4 or 5) vs. those who were neutral or disagreed (score of 1, 2 or 3).

### Statistics

The primary aim was descriptive. The secondary aim evaluated the adaptation decision made for each of the statements on the symptom management care pathway templates and did not include new statements added by the institution. This outcome was dichotomized as reject vs. did not reject for each statement. The institutional factors evaluated against adaptation choice were informed by CFIR domain constructs [18, 19] and other institutional contextual variables as follows: number of pediatric patients with cancer diagnosed annually, proportion of patients with private insurance, median physician years in practice and median nurse practitioner years in practice. These factors were dichotomized as high or low at the median value across the 10 institutions. We also planned to evaluate the CFIR inner setting measures focused on implementation climate dichotomized as agree vs. did not agree. These were as follows: staff are expected to help the institution meet its goal, staff receives the support they need to implement care pathways for symptom management, staff receives recognition for implementing care pathways for symptom management and implementing care pathways for symptom management is a top priority of the department.

The analysis consisted of a generalized linear mixed model treating statement and institution as random effects and the covariate of interest as a fixed effect. P < 0.05 was considered statistically significant. Analyses were performed using R studio version 3.6.1, The R Foundation for Statistical Computing.

## Results

Table 1 illustrates the demographic characteristics of the 10 institutions and Additional file 3 shows baseline CPG and care pathway utilization. The median number of pediatric patients diagnosed annually was 98 (interquartile range (IQR) 64 to 180), the median percentage of patients with private insurance was 44 (IQR 26 to 59), the median physician years in practice (all physicians, not restricted to the institutional adaptation team) was 15 (IQR 10 to 19) and the median nurse practitioner years in practice was 7 (IQR 4 to 8). Table 1 also shows the results of the implementation climate construct of the CFIR. All 10 institutions agreed that department staff were expected to help the institution meet its goal of improving symptom control for their patients while 6 agreed they receive support they need to implement symptom management care pathways, 5 agreed they receive recognition for their implementation and 6 agreed that their implementation was a top priority of the department.

**Table 1:** Characteristics of Institutions (N = 10)

	Value
Patient Population Characteristic	
Pediatric vs. Mixed Adult and Pediatric, n (%)	7 (70.0)
Median Number Pediatric Patients with Cancer Diagnosed Annually (IQR)	98 (64 to 180)
Median Insurance Type Percentage (IQR)	
Private	44 (26 to 59)
Public	56 (41 to 73)
No insurance	1 (0 to 4)
Median Male Percentage (IQR)	54 (51 to 55)
Median Race Percentage (IQR)	
American Indian or Alaskan native	0 (0 to 1)
Asian	9 (3 to 10)
Black or African American	9 (6 to 20)
Native Hawaiian or other pacific islander	1 (0 to 3)
White	69 (41 to 80)
Median Hispanic or Latino Ethnicity (IQR)	28 (10 to 42)
Median Language Spoken Percentage (IQR)	
English	78 (64 to 85)
Spanish	14 (4 to 32)
Other	5 (2 to 5)
**Health care Professional Characteristics**	
Physician Characteristics, median (IQR)	
Full-time equivalents	8 (6 to 11)
Years in practice	15 (10 to 19)
Nurse Practitioner Characteristics	
Full-time equivalents	2 (2 to 11)
Years in practice	7 (4 to 8)
Physician Assistant Characteristics	
Full-time equivalents	1 (0 to 1)
Years in practice	2 (0 to 5)
**Implementation Climate Construct of Consolidated Framework for Implementation Research Framework**	
Department staff are expected to help the institution meet its goal (i.e., improve symptom control for patients), n (%)	10 (100)
Department staff gets the support they need to implement care pathways for symptom management, n (%)	6 (60.0)
Department staff gets recognition for implementing care pathways for symptom management, n (%)	5 (50.0)
Implementing care pathways for symptom management is a top priority of the department, n (%)	6 (60.0)

Abbreviation: IQR - interquartile range

Figure 1 shows the time required to accomplish each phase of the symptom management care pathway adaptation process. During the preparation phase, two institutions asked for a third co-lead, resulting in 22 institutional adaptation team members. The professions of institutional adaptation team members were as follows: physician (n = 16), nurse or nurse practitioner (n = 5) and pharmacist (n = 1). The median time to accomplish the initial care pathway adaptation decision-making phase was 4.2 weeks (IQR 2.0 to 5.3) per institution. During this phase, none of the 10 institutions requested an adaptation panel be convened.

Table 2 shows that across all 10 institutions, the number of statements for prevention, assessment and treatment were 570 (42.2%), 60 (4.4%) and 720 (53.3%), respectively. The number of statements per symptom ranged from 2 (anger and taste changes) to 25 (pain). In total, there were 135 statements across all 14 symptom management care pathways and 1350 decisions were made by the 10 institutions. Across all institutions, most statements were adopted or adapted (1,208/1350 (89.5%) while 86 (6.4%) statements were rejected (Table 2). The proportion of statements rejected by each institution ranged from 3.0% to 15.6%. Table 2 also describes specific statement adaptations and reasons for rejections. Most commonly, the reason for rejection was not agreeing with the statement (70/86, 81.4%). Additional file 4 shows the distribution of adaptation decisions by symptom. Statements were rejected most commonly for the mucositis care pathway.

**Table 2:** Distribution of Statements and Adaptation Choices across 10 Institutions (N = 1350 Statements*)

Variables	n (%)
Section	
Prevention	570 (42.2)
Assessment	60 (4.4)
Treatment	720 (53.3)
Adaptation Choice	
Adopt	551 (40.8)
Adapt	657 (48.7)
Reject	86 (6.4)
Because of previous decision, statement no longer applicable	56 (4.1)
New statement added*	65
Adaptations**	n = 657
Resource(s) added	96 (14.6)
Resource(s) partially removed (some retained)	134 (20.4)
Resource(s) all removed (none retained)	14 (2.1)
Added statement to follow institutional standard or order set	32 (4.9)
Removed statement to follow institutional standard	13 (2.0)
Articulated action be based on severity of bothersome symptom	216 (32.9)
Changed conditional (“consider x”) to strong (“do x”) statement	146 (22.2)
Changed content of statement or combined two or more statements	261 (39.7)
Medication(s) removed	65 (9.9)
Medication(s) added	48 (7.3)
Medication substituted with a related medication	50 (7.6)
Statement moved to another section (e.g. prevention to treatment)	11 (1.7)
Removed or modified hyperlink	21 (3.2)
Rejections	n = 86
Statement not relevant for primary team	8 (9.3)
Did not agree with statement	70 (81.4)
Personnel unable to offer intervention due to lack of availability	8 (9.3)

* Denominator for adaptation choice was the number of statements on the template care pathways (N = 1350); new statements added were not included in this denominator

** For adaptations, there could be multiple adaptations per statement

Table 3 describes how adaptation decisions were distributed by prevention, assessment and treatment sections. Rejections were most common in the prevention section (47/570, 8.2%) and least common in the assessment section (1/60, 1.7%). Adaptation consisting of change in content was most common in the treatment section (152/450, 33.8%).

**Table 3:** Distribution of Adaptation Choice by Section across 10 Institutions (N = 1350 Statements*)

	Prevention	Assessment	Treatment
	n (%)	n (%)	n (%)
N	570	60	720
Adaptation Choice			
Adopt	301 (52.8)	29 (48.3)	221 (30.7)
Adapt	177 (31.1)	30 (50.0)	450 (62.5)
Reject	47 (8.2)	1 (1.7)	38 (5.3)
Because of previous decision, statement no longer applicable	45 (7.9)	0 (0.0)	11 (1.5)
Adaptations	n = 177	n = 30	n = 450
Resource(s) added	14 (7.9)	6 (20.0)	76 (16.9)
Resource(s) partially removed (some retained)	15 (8.5)	0 (0.0)	119 (26.4)
Resource(s) all removed (none retained)	1 (0.6)	0 (0.0)	13 (2.9)
Added statement to follow institutional standard or order set	10 (5.6)	6 (20.0)	16 (3.6)
Removed statement to follow institutional standard	1 (0.6)	1 (3.3)	11 (2.4)
Articulated action be based on severity of bothersome symptom	19 (10.7)	11 (36.7)	186 (41.3)
Changed conditional (“consider x”) to strong (“do x”) statement	44 (24.9)	1 (3.3)	101 (22.4)
Changed content of statement or combined two or more statements	97 (54.8)	12 (40.0)	152 (33.8)
Medication(s) removed	14 (7.9)	0 (0.0)	51 (11.3)
Medication(s) added	16 (9.0)	0 (0.0)	32 (7.1)
Medication substituted with a related medication	0 (0.0)	0 (0.0)	50 (11.1)
Statement moved to another section (e.g. prevention to treatment)	2 (1.1)	1 (3.3)	8 (1.8)
Removed or modified hyperlink	13 (7.3)	0 (0.0)	8 (1.8)
Rejections	n = 47	n = 1	n = 38
Statement not relevant for primary team	3 (6.4)	1 (100.0)	4 (10.5)
Did not agree with statement	42 (89.4)	0 (0.0)	28 (73.7)
Personnel unable to offer intervention due to lack of availability	2 (4.3)	0 (0.0)	6 (15.8)

* Denominator for adaptation choice was the number of statements on the template care pathways (N = 1350); new statements added were not included in this denominator

Table 4 shows the distribution of adaptation decisions by the institutional factors evaluated and the results of the generalized linear mixed model where the outcome was reject vs. not reject. In the model with only statements and institutions, variation was greater between statements (variance = 43.2) than between institutions (variance = 0.8). To model whether a covariate or institutional factor was associated with the outcome of reject vs. not reject, there must be variation in the covariate (since otherwise, there would be no comparison group). For this reason, we could not evaluate department staff are expected to help the institution meet its goal, as all 10 institutions agreed with this statement. Table 4 also shows that none of the institutional factors was significantly associated with statement rejection.

**Table 4:** Institutional Factors Associated with Adaptation Choice

Characteristics*	Adopt	Adapt	Reject	Estimate ± SE	P Value**
	N = 551	N = 657	N = 86		
	n (%)	n (%)	n (%)		
Volume Pediatric Patients with Cancer Diagnosed Annually				-0.57 ± 0.63	0.369
High	272 (49.4)	335 (51.0)	36 (41.9)		
Low	279 (50.6)	322 (49.0)	50 (58.1)		
Proportion of Patients with Private Insurance				-0.86 ± 0.61	0.162
High	277 (50.2)	336 (51.1)	34 (39.5)		
Low	274 (49.7)	321 (48.9)	52 (60.5)		
Median Physician Years in Practice (IQR)				-1.00 ± 0.59	0.091
High	302 (54.8)	330 (50.2)	33 (38.4)		
Low	249 (45.2)	327 (49.8)	53 (61.6)		
Median Nurse Practitioner Years in Practice (IQR)				-1.00 ± 0.60	0.095
High	330 (59.9)	403 (61.3)	41 (47.7)		
Low	221 (40.1)	254 (38.7)	45 (52.3)		
Receive Support to Implement Care Pathways for Symptom Management				-0.71 ± 0.64	0.272
Agree	336 (61.0)	389 (59.2)	59 (68.6)		
Disagree	215 (39.0)	268 (40.8)	27 (31.4)		
Receive Recognition for Implementing Care Pathways for Symptom Management				0.78 ± 0.62	0.207
Agree	287 (52.1)	331 (50.4)	35 (40.7)		
Disagree	264 (47.9)	326 (49.6)	51 (59.3)		
Implementing Care Pathways for Symptom Management is a Top Priority of the Department				-0.59 ± 0.65	0.359
Agree	328 (59.5)	391 (59.5)	58 (67.4)		
Disagree	223 (40.5)	226 (40.5)	28 (32.6)		

* Characteristics dichotomized at median value for the 10 institutions

** P value derived from a generalized linear mixed model treating statement and institution as random effects and the covariate of primary interest as a fixed effect where outcome was reject vs. no reject

Abbreviations: IQR – interquartile range; SE – standard error

Additional file 5 shows the results of the implementation survey. All principal investigators chose to distribute emails themselves and thus, number of emails distributed was not captured. There were 84 respondents to the survey across institutions. The care pathways most likely to be used were mucositis, pain and nausea and vomiting while the care pathways least likely to be used were body changes, taste changes and anger.

## Discussion

We created 14 pediatric cancer symptom management care pathway templates based on CPGs and developed a process to adapt them at 10 institutions. We found that most care pathway statements were adopted or adapted, with 6.4% of statements being rejected. Most commonly, the reason for statement rejection was not agreeing with the statement. We also found that that this process could be accomplished at each institution within a relatively short timeframe.

Our description of the care pathway template development and adaptation process is important because implementation of institutional care pathways will likely be an important step toward optimizing supportive care delivery and may result in more consistent care delivery within and across institutions [22, 23]. Implementation of institutional care pathways may also allow healthcare professionals to feel more confident about delivery of care and may empower them to be proactive in identifying and addressing deviations from their care pathway.

We do not claim that this process is the only approach toward care pathway development and adaptation and the process will most likely evolve and improve as experience grows. However, the description of this process may facilitate care pathway implementation across institutions in multiple supportive care areas. While our process was successful, it did require considerable resources to enable real-time care pathway adaptations through a series of videoconference calls. It is possible that providing the initial templates to institutions and having them perform the adaptation process independently might be a feasible approach that is less resource intensive. This approach would likely benefit from a local champion to lead the adaptation process and provision of training to local champions. Software to facilitate this process also may be useful.

There may be challenges to care pathway implementation [24–29]. We included an interprofessional review stage in our procedures to facilitate implementation. Rotter et al. noted that implementation strategies that do not involve the relevant parties may adversely affect the impact of care pathways [23]. The inclusion of key representatives in the care pathway review phase may lead to more successful utilization. Nonetheless, care pathway uptake and utilization are likely to be challenging.

The key outcomes of this process have yet to be measured. They include whether clinicians provide care consistent with their institutional care pathways, and whether patient outcomes are improved when care pathway-consistent care is delivered. These outcomes will be measured and reported as part of the cluster randomized trial. However, as more institutions strive to develop and implement care pathways for supportive care, measuring these outcomes more widely would be useful to improve our fundamental understanding of how these interventions can and should be implemented. It is likely that multi-faceted interventions to improve care pathway implementation will be required.

A strength of this work is its conduct at a relatively large number of institutions, providing reassurance that this process can be instituted across different types of institutions. Another strength is the careful documentation of adaptation decisions and the ability to compare these decisions across symptom management care pathways and across institutions. However, our results are limited by the conduct of this study only at institutions in the United States. It is possible that results may differ in other countries.

## Conclusions

In conclusion, acceptability of the 14 care pathways was evident by most statements being adopted or adapted. The adaptation process for 14 care pathways was accomplished in a relatively short timeframe, with a median time to accomplish the initial care pathway adaptation decision-making phase over 4.2 weeks per institution. The proportion of statements rejected by each institution ranged from 3.0% to 15.6%. Most commonly, the reason for rejection was not agreeing with the statement. Future work should focus on evaluation of care pathway compliance and determination of the impact of care pathway-consistent care on patient outcomes.

## Electronic supplementary material

Below is the link to the electronic supplementary material.

**Additional file 1**: Care Pathway Template Example

**Additional file 2**: Options for Adaptations Considering Degree of Bothersome Symptoms

**Additional file 3**: Clinical Practice Guideline and Care Pathway Utilization for Symptom Management at Baseline (N=10)

**Additional file 4**: Distribution of Sections and Adaptation Choices by Symptom Care Pathway

**Additional file 5**: Results of the Implementation Survey to all Healthcare Professionals across Institutions (N=84)

## Data Availability

The datasets used and/or analyzed during the current study are available from the corresponding author on reasonable request.

## Abbreviations

CPG: Clinical practice guideline
SPARK: Supportive care Prioritization, Assessment and Recommendations for Kids
SSPedi: Symptom Screening in Pediatrics Tool
CFIR: Consolidated Framework for Implementation Research
IQR: Interquartile range

## References

[1] Alexander S, Pole JD, Gibson P, Lee M, Hesser T, Chi SN (2015). Classification of treatment-related mortality in children with cancer: a systematic assessment. Lancet Oncol.

[2] Lehrnbecher T, Ethier MC, Zaoutis T, Creutzig U, Gamis A, Reinhardt D (2009). International variations in infection supportive care practices for paediatric patients with acute myeloid leukaemia. Br J Haematol.

[3] Loeffen EA, Mulder RL, van de Wetering MD, Font-Gonzalez A, Abbink FC, Ball LM (2016). Current variations in childhood cancer supportive care in the Netherlands. Cancer.

[4] Institute of Medicine (US) Committee on Standards for Developing Trustworthy Clinical Practice Guideline. Clinical Practice Guidelines We Can Trust Washington (DC): National Academies Press (US); [Available from: https://www.ncbi.nlm.nih.gov/books/NBK209539/

[5] Dupuis LL, Robinson PD, van de Wetering MD, Tissing W, Seelisch J, Digout C (2020). Lexicon for guidance terminology in pediatric hematology/oncology: a White Paper. Pediatr Blood Cancer.

[6] Clinical Practice Guidelines (1990). Directions for a New Program, Institute of Medicine.

[7] Clinical Practice Guidelines We Can Trust. Washington, D.C.:Institute of Medicine, National Academies Press; 2011. 24983061

[8] Guyatt GH, Oxman AD, Vist GE, Kunz R, Falck-Ytter Y, Alonso-Coello P (2008). GRADE: an emerging consensus on rating quality of evidence and strength of recommendations. BMJ.

[9] Shrank WH, Rogstad TL, Parekh N (2019). Waste in the US Health Care System: estimated costs and potential for savings. JAMA.

[10] Campbell H, Hotchkiss R, Bradshaw N, Porteous M (1998). Integrated care pathways. BMJ.

[11] Cook S, Vettese E, Soman D, Hyslop S, Kuczynski S, Spiegler B (2019). Initial development of supportive care Assessment, prioritization and recommendations for kids (SPARK), a symptom screening and management application. BMC Med Inform Decis Mak.

[12] Vettese E, Cook S, Soman D, Kuczynski S, Spiegler B, Davis H (2019). Longitudinal evaluation of supportive care prioritization, Assessment and Recommendations for kids (SPARK), a symptom screening and management application. BMC Cancer.

[13] Dupuis LL, Johnston DL, Baggott C, Hyslop S, Tomlinson D, Gibson P (2018). Validation of the Symptom Screening in Pediatrics Tool in Children receiving Cancer treatments. J Natl Cancer Inst.

[14] O’Sullivan C, Dupuis LL, Gibson P, Johnston DL, Baggott C, Portwine C (2014). Refinement of the symptom screening in pediatrics tool (SSPedi). Br J Cancer.

[15] Tomlinson D, Robinson PD, Gibson P, Beauchemin M, Grimes A, Dadzie G et al. Creating and adapting an infection management care pathway in pediatric oncology.Support Care Cancer. 2022. 10.1007/s00520-022-07216-x 35731317

[16] National Guideline Clearinghouse. Inclusion Criteria. http://www.guideline.gov/about/inclusion-criteria.aspx 2014 [

[17] Dupuis LL, Grimes A, Vettese E, Klesges LM, Sung L (2021). Barriers to symptom management care pathway implementation in pediatric Cancer. BMC Health Serv Res.

[18] Damschroder LJ, Aron DC, Keith RE, Kirsh SR, Alexander JA, Lowery JC (2009). Fostering implementation of health services research findings into practice: a consolidated framework for advancing implementation science. Implement Sci.

[19] Fernandez ME, Walker TJ, Weiner BJ, Calo WA, Liang S, Risendal B (2018). Developing measures to assess constructs from the Inner setting domain of the Consolidated Framework for implementation research. Implement Sci.

[20] Helfrich CD, Li YF, Mohr DC, Meterko M, Sales AE (2007). Assessing an organizational culture instrument based on the competing values Framework: exploratory and confirmatory factor analyses. Implement Sci.

[21] Shortell SM, Zazzali JL, Burns LR, Alexander JA, Gillies RR, Budetti PP (2001). Implementing evidence-based medicine: the role of market pressures, compensation incentives, and culture in physician organizations. Med Care.

[22] Rotter T, Kinsman L, James E, Machotta A, Gothe H, Willis J et al. Clinical pathways: effects on professional practice, patient outcomes, length of stay and hospital costs.Cochrane Database Syst Rev. 2010(3):CD006632. 10.1002/14651858.CD006632.pub2 20238347

[23] Rotter T, de Jong RB, Lacko SE, Ronellenfitsch U, Kinsman L (2019). Clinical pathways as a quality strategy. Improving healthcare quality in Europe: characteristics, effectiveness and implementation of different strategies.

[24] Bierbaum M, Rapport F, Arnolda G, Delaney GP, Liauw W, Olver I (2022). Clinical practice guideline adherence in oncology: a qualitative study of insights from clinicians in Australia. PLoS ONE.

[25] Rosa RG, Teixeira C, Sjoding M (2020). Novel approaches to facilitate the implementation of guidelines in the ICU. J Crit Care.

[26] Brennan C, Greenhalgh J, Pawson R (2018). Guidance on guidelines: understanding the evidence on the uptake of health care guidelines. J Eval Clin Pract.

[27] Scott IA, Denaro CP, Bennett CJ, Mudge AM (2004). Towards more effective use of decision support in clinical practice: what the guidelines for guidelines don’t tell you. Intern Med J.

[28] Correa VC, Lugo-Agudelo LH, Aguirre-Acevedo DC, Contreras JAP, Borrero AMP, Patino-Lugo DF (2020). Individual, health system, and contextual barriers and facilitators for the implementation of clinical practice guidelines: a systematic metareview. Health Res Policy Syst.

[29] Sugalski AJ, Lo T, Beauchemin M, Grimes AC, Robinson PD, Walsh AM (2021). Facilitators and barriers to clinical practice guideline-consistent supportive care at pediatric oncology institutions: a Children’s Oncology Group study. Implement Sci Commun.

