# Supplementary figures and images for: Symptom management care pathway adaptation process and specific adaptation decisions
Source: BMC Cancer. 2023 Apr 17;23:350. doi: 10.1186/s12885-023-10835-0 (PMC10108500; doi:10.1186/s12885-023-10835-0)

**Additional file 1: Care Pathway Template Example**


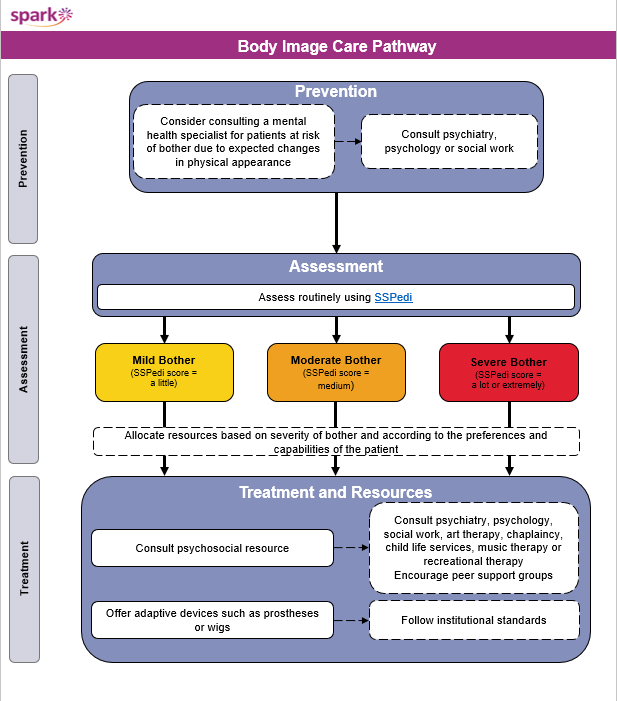

Supplement: Supplementary file 1 — Additional file 1: Care Pathway Template Example [file 12885_2023_10835_MOESM1_ESM.docx]
