# Supplementary material for: Symptom management care pathway adaptation process and specific adaptation decisions
Source: BMC Cancer. 2023 Apr 17;23:350. doi: 10.1186/s12885-023-10835-0 (PMC10108500; doi:10.1186/s12885-023-10835-0)
Supplement: Supplementary file 2 — Additional file 2: Options for Adaptations Considering Degree of Bothersome Symptoms [file 12885_2023_10835_MOESM2_ESM.docx]

**Additional file 2: Options for Adaptations Considering Degree of Bothersome Symptoms**

Original Template

**
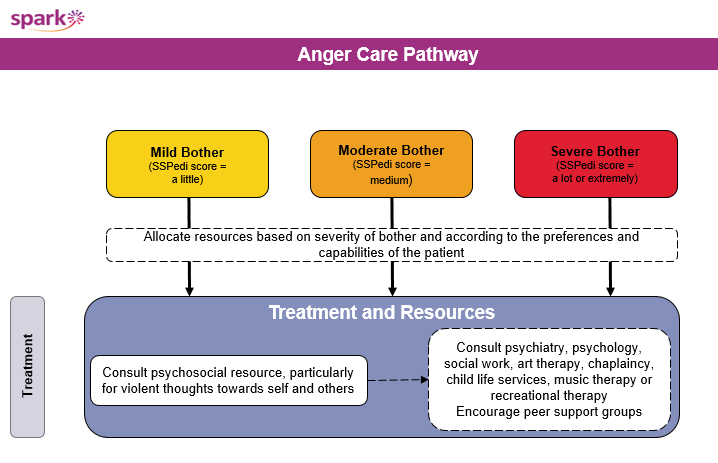
**

Option 2

Option 1

**
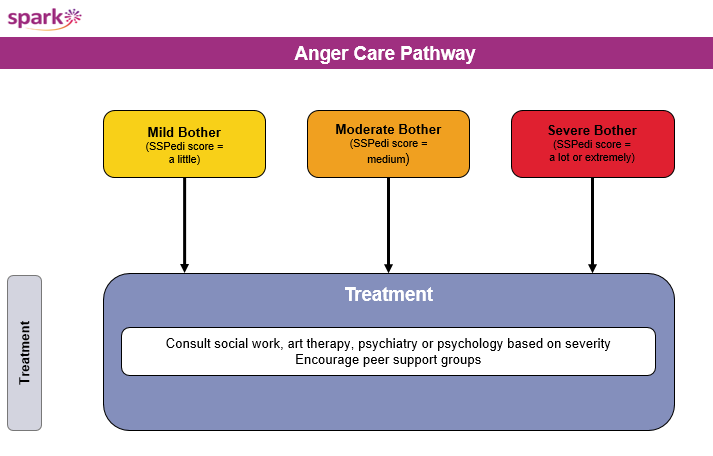

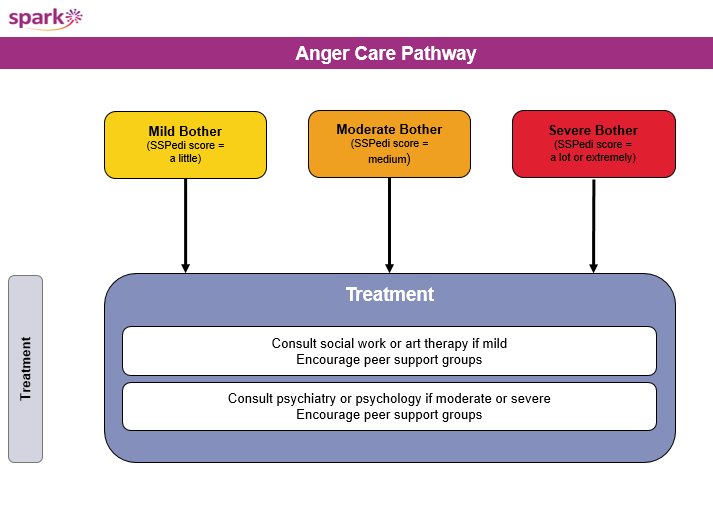
**
