# Supplementary material for: Symptom management care pathway adaptation process and specific adaptation decisions
Source: BMC Cancer. 2023 Apr 17;23:350. doi: 10.1186/s12885-023-10835-0 (PMC10108500; doi:10.1186/s12885-023-10835-0)
Supplement: Supplementary file 3 — Additional file 3: Clinical Practice Guideline and Care Pathway Utilization for Symptom Management at Baseline (N=10) [file 12885_2023_10835_MOESM3_ESM.docx]

**Additional file 3: Clinical Practice Guideline and Care Pathway Utilization for Symptom Management at Baseline (N=10)**

| **Variables** | **n (%)** |
| --- | --- |
| **Institution Use of CPGs** | 6 (60%) |
| Symptoms for which Institution Uses CPGs |  |
| Nausea and vomiting | 5 (50%) |
| Anorexia | 3 (30%) |
| Constipation | 3 (30%) |
| Pain | 2 (20%) |
| Fatigue | 1 (10%) |
| Extent CPGs Routinely Used by Healthcare Professionals |  |
| Never | 0 |
| A little | 0 |
| Somewhat | 1 (10%) |
| A lot | 4 (40%) |
| Always | 1 (10%) |
|  |  |
| **Institution Use of Care Pathways** | 6 (60%) |
| Symptoms for Which Institution Uses Care Pathways |  |
| Nausea and vomiting | 5 (50%) |
| Pain | 2 (20%) |
| Anorexia | 1 (10%) |
| Constipation | 1 (10%) |
| Fatigue | 1 (10%) |
| Extent Care Pathways are Routinely Used by Healthcare Professionals |  |
| Never | 0 |
| A little | 0 |
| Somewhat | 1 (10%) |
| A lot | 4 (40%) |
| Always | 1 (10%) |

Abbreviation: CPG – clinical practice guideline
