# Supplementary material for: Symptom management care pathway adaptation process and specific adaptation decisions
Source: BMC Cancer. 2023 Apr 17;23:350. doi: 10.1186/s12885-023-10835-0 (PMC10108500; doi:10.1186/s12885-023-10835-0)
Supplement: Supplementary file 4 — Additional file 4: Distribution of Sections and Adaptation Choices by Symptom Care Pathway [file 12885_2023_10835_MOESM4_ESM.docx]

**Additional file 4: Distribution of Sections and Adaptation Choices by Symptom Care Pathway**

|  | Anger | Anxiety | Appetite Changes | Body Changes | Cognition | Constipation | Depression | Diarrhea | Fatigue | Mucositis | Nausea and Vomiting | Pain | Peripheral Neuropathy | Taste Changes |
| --- | --- | --- | --- | --- | --- | --- | --- | --- | --- | --- | --- | --- | --- | --- |
| **Number Statements** | **20** | 30 | 50 | 30 | 30 | 130 | 100 | 150 | 100 | 100 | 240 | 250 | 100 | 20 |
|  | n (%) | n (%) | n (%) | n (%) | n (%) | n (%) | n (%) | n (%) | n (%) | n (%) | n (%) | n (%) | n (%) | n (%) |
| Section |  |  |  |  |  |  |  |  |  |  |  |  |  |  |
| Prevention | 10 (50.0) | 10 (33.3) | 10 (20.0) | 10 (33.3) | 10 (33.3) | 20 (15.4) | 20 (20.0) | 20 (13.3) | 40 (40.0) | 60 (60.0) | 170 (70.8) | 170 (68.0) | 10 (10.0) | 10 (50.0) |
| Assessment | 0 (0.0) | 0 (0.0) | 10 (20.0) | 0 (0.0) | 0 (0.0) | 10 (7.7) | 10 (10.0) | 20 (13.3) | 0 (0.0) | 10 (10.0) | 0 (0.0) | 0 (0.0) | 0 (0.0) | 0 (0.0) |
| Treatment | 10 (50.0) | 20 (66.7) | 30 (60.0) | 20 (66.7) | 20 (66.7) | 100 (76.9) | 70 (70.0) | 110 (73.3) | 60 (60.0) | 30 (30.0) | 70 (29.2) | 80 (32.0) | 90 (90.0) | 10 (50.0) |
| Adaptation Choice |  |  |  |  |  |  |  |  |  |  |  |  |  |  |
| Keep as is | 9 (45.0) | 17 (56.7) | 17 (34.0) | 2 (6.7) | 17 (56.7) | 37 (28.5) | 29 (29.0) | 62 (41.3) | 36 (36.0) | 43 (43.0) | 129 (53.8) | 94 (37.6) | 46 (46.0) | 13 (65.0) |
| Adapt | 11 (55.0) | 12 (40.0) | 28 (56.0) | 28 (93.3) | 12 (40.0) | 91 (70.0) | 65 (65.0) | 75 (50.0) | 51 (51.0) | 31 (31.0) | 64 (26.7) | 134 (53.6) | 48 (48.0) | 7 (35.0) |
| Reject | 0 (0.0) | 1 (3.3) | 1 (2.0) | 0 (0.0) | 0 (0.0) | 0 (0.0) | 6 (6.0) | 10 (6.7) | 13 (13.0) | 26 (26.0) | 18 (7.5) | 6 (2.4) | 5 (5.0) | 0 (0.0) |
| No longer applicable | 0 (0.0) | 0 (0.0) | 4 (8.0) | 0 (0.0) | 1 (3.3) | 2 (1.5) | 0 (0.0) | 3 (2.0) | 0 (0.0) | 0 (0.0) | 29 (12.1) | 16 (6.4) | 1 (1.0) | 0 (0.0) |
