# Supplementary material for: Symptom management care pathway adaptation process and specific adaptation decisions
Source: BMC Cancer. 2023 Apr 17;23:350. doi: 10.1186/s12885-023-10835-0 (PMC10108500; doi:10.1186/s12885-023-10835-0)
Supplement: Supplementary file 5 — Additional file 5: Results of the Implementation Survey to all Healthcare Professionals across Institutions (N=84) [file 12885_2023_10835_MOESM5_ESM.docx]

**Additional file 5: Results of the Implementation Survey to all Healthcare Professionals across Institutions (N=84)**

| **Variable** | **n (%)** |
| --- | --- |
| Profession |  |
| Art therapist | 1 (1%) |
| Chaplain | 2 (2%) |
| Child life services | 4 (5%) |
| Contact nurse | 11 (13%) |
| Music therapist | 0 (0%) |
| Nurse educator | 5 (6%) |
| Nutrition services | 2 (2%) |
| Occupational therapist | 1 (1%) |
| Oncologist | 28 (33%) |
| Pharmacist | 2 (2%) |
| Physical therapist | 2 (2%) |
| Psychiatrist | 0 |
| Psychologist | 4 (5%) |
| Recreational therapist | 0 |
| Social worker | 5 (6%) |
| Other* | 17 (20%) |
| Care Pathway Most Likely to Use** |  |
| Mucositis | 54/65 (83%) |
| Pain | 60/78 (77%) |
| Nausea and vomiting | 60/79 (73%) |
| Anxiety | 57/79 (72%) |
| Constipation | 51/72 (71%) |
| Appetite changes | 46/65 (70%) |
| Diarrhea | 41/63 (65%) |
| Fatigue | 53/82 (65%) |
| Peripheral neuropathy | 51/79 (65%) |
| Depression | 48/84 (57%) |
| Body changes | 40/79 (51%) |
| Anger | 36/79 (46%) |
| Cognition | 31/68 (46%) |
| Taste changes | 27/65 (42%) |
| Care Pathway Least Likely to Use** |  |
| Body changes | 29/79 (37%) |
| Taste changes | 22/65 (34%) |
| Anger | 23/79 (29%) |
| Cognition | 13/68 (19%) |
| Constipation | 13/72 (18%) |
| Peripheral neuropathy | 14/79 (18%) |
| Nausea and vomiting | 13/79 (16%) |
| Depression | 13/84 (15%) |
| Fatigue | 9/82 (11%) |
| Anxiety | 10/79 (13%) |
| Diarrhea | 6/63 (10%) |
| Mucositis | 5/65 (7%) |
| Appetite changes | 4/65 (6%) |
| Pain | 5/78 (6%) |

* Other profession: registered nurse, nurse practitioner, clinical nurse coordinator, clinical rehabilitation supervisor, physician assistant, transplant and cell therapy

** Not all care pathways were shown to all respondents – only relevant care pathways were shown based upon their self-declared healthcare professional type
